# On the Occasional Bad Effects of Ergot, Administered to Premote Parturition

**Published:** 1814-09

**Authors:** 


*For the Medical and Physical Journal.*

### *On the occasional bad Effects of Ergot, administered to promote Parturition.*

HAVING observed by some publications, that suspicions and doubts have arisen as to the occasional bad effects of Ergot on the infant during parturition, I beg leave to state the following case.

Some time since, I was requested to attend a patient, then in labour for the third time. Her two former labours had been perfectly natural; the second especially was attended with very few pains. When I visited her, the pains were rather strong, and the os uteri so well dilated, that a very speedy termination of the labour seemed probable. Soon after, the os uteri became fully dilated; but in a short time I observed that the head advanced more slowly than one might expect under such circumstances. The pains, however, continued as long as two hours more, then gradually subsided, and at last wholly ceased. Various attempts were made to re-excite the pains by stimulating the os uteri with moderate pressure, by causing the patient to rise and walk about, yet all without effect. Conceiving that further delay might be attended with some ill consequence, I resolved to employ the lever, having a confidence in that instrument, and a facility in the use of it. In a short time the head was moved from the spot where it seemed to be bedded, and the operation of the instrument being aided by slight pains, in the space of twenty-five minutes a healthy child was delivered.

On

On drawing down the umbilical cord, with the intention of extracting the placenta, more than common resistance was felt, which induced me to place a hand on the abdomen, by which it appeared there was another child to be delivered. This circumstance was not mentioned to the mother, as it was thought likely to discourage her, after the suffering and apprehension she had already experienced; but she was told that it would be necessary to wait an hour for the extraction of the placenta. The hour passed away therefore with great patience on the part of the mother, but not so much on mine; for there was not the slightest appearance of pains during that time. At this moment I was called on by another practitioner, to whom I thought proper to mention the case, when he suggested the use of Ergot. Having myself sometimes used that substance, I was surprised that it had not occurred to me in this instance, and immediately resolved to employ it. Fifteen grains of the powder were at once given in a little water. In fifteen or twenty minutes the pains came on, and continued without remission till the child was born, which was in about twenty minutes from the time the pains commenced, the head being born first, as in natural labours. The child made no efforts to cry, which induced me to dash water and spirit upon it, then to rub it with the latter about the head and breast, and to clear its mouth of mucus; these things failing, I attempted to inflate the lungs, but some time being consumed in obtaining the necessary articles, the child was lost. It was in every respect as fine a child as the first, perfectly fresh and firm; and the umbilical cord pulsated when it was first born.

Every one who is acquainted with the facility with which, in a case of twins, the second child makes its way into the world, will consider the death of the child, in this instance, as an unusual occurrence. For my part, I confess I was at first wholly at a loss to explain it; but on mentioning it to two or three medical friends, one gentleman remarked that his observations had led him to believe that *Ergot* sometimes proved fatal to the child; and another, a physician of great experience, said, without any intimation of the kind from me, "Ergot kills the children." On what observations the opinions of these gentlemen were founded I know not; but, since I have reflected on the matter, have no hesitation in declaring my opinion, that in the case related above, the unremitting contraction of the uterus, produced by the administration of this substance, forced down the child so steadily and strongly, as by a long-continued and violent pressure on the brain to destroy the life of the child.

A YOUNG PRACTITIONER.

*(New-Engl. Journal.)*

